# Spatial Mass Spectrometry-Based Proteomic Analysis of Normal-Appearing Glomeruli from Young and Old Adults

**DOI:** 10.34067/KID.0000000986

**Published:** 2025-09-30

**Authors:** Kiran K. Mangalaparthi, Gunveen S. Sachdeva, Afsana Ansari Shaik, Shilpa Venkataraman, Aidan F. Mullan, Ganesh P. Pujari, Benjamin J. Madden, Muhammad Sohaib Asghar, Vidit Sharma, Mariam P. Alexander, Nicholas B. Larson, Aleksandar Denic, Akhilesh Pandey, Andrew D. Rule

**Affiliations:** 1 Department of Laboratory Medicine and Pathology, Mayo Clinic, Rochester, Minnesota; 2 Manipal Academy of Higher Education, Manipal, India; 3 Division of Nephrology and Hypertension, Mayo Clinic, Rochester, Minnesota; 4 Department of Quantitative Health Sciences, Mayo Clinic, Rochester, Minnesota; 5 Proteomics Core, Mayo Clinic, Rochester, Minnesota; 6 Department of Urology, Mayo Clinic, Rochester, Minnesota; 7 Center for Individualized Medicine, Mayo Clinic, Rochester, Minnesota; 8 Division of Epidemiology, Mayo Clinic, Rochester, Minnesota

**Keywords:** CKD, extracellular matrix, glomerular disease, renal function decline

## Abstract

**Key Points:**

- Increased protein expression of tissue inhibitor of metalloproteinase-3, glypican 6, synuclein *γ*, apolipoprotein A-4, and Ecto-5′-nucleotidase was observed in old adults compared with young adults.
- In old adults, overexpressed proteins were enriched in mitochondrial processes while under expressed proteins enriched in RNA metabolism.
- Validated differentially expressed proteins were not significantly correlated with kidney pathology after adjusting for age.

**Background:**

Kidney aging is characterized by a loss of glomeruli, predominantly in the superficial cortex, with a resultant decline in GFR and an increased risk of various kidney-related diseases. The early molecular alterations in glomeruli associated with the aging process are not well studied.

**Methods:**

We combined laser capture microdissection and mass spectrometry–based unbiased proteomic analysis of nonsclerosed, nonischemic glomeruli in the superficial cortex from young and old adults who underwent a radical nephrectomy for a tumor to understand the age-related molecular changes in glomeruli. Twenty-four young and 30 old adults were used for the discovery dataset, and the significant differentially expressed proteins were further validated using an independent set comprising six young and eight old adults.

**Results:**

Kidneys from old adults had lower eGFR, less kidney parenchyma on computed tomography imaging, and more glomerulosclerosis and arteriosclerosis on histology. Quantitative proteomic analysis of nonsclerosed, nonischemic glomeruli identified increased expression of tissue inhibitor of metalloproteinase-3, glypican 6, synuclein *γ*, apolipoprotein A-4, and Ecto-5′-nucleotidase in old adults that were further validated in an independent set. Pathway analysis indicated that proteins with increased expression in old adults were enriched in mitochondrial translational processes, aerobic respiration, and TCA cycle, whereas proteins with decreased expression in old adults were enriched in mRNA splicing, mRNA processing, and nonsense mediated decay. Furthermore, validated differentially expressed proteins did not show any significant correlation with the kidney pathology independent of age group.

**Conclusions:**

Overall, this study identified proteins that are specifically associated with the aging process in otherwise normal-appearing glomeruli.

## Visual Abstract

**Figure:**
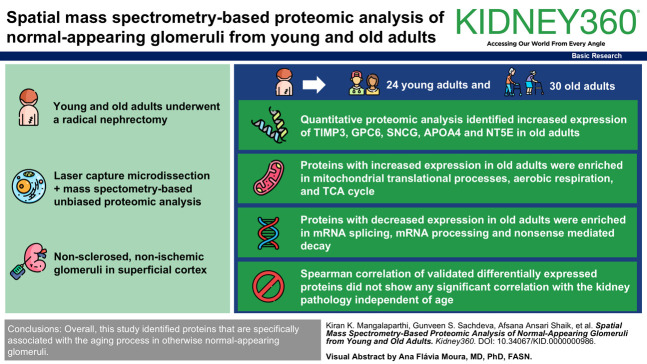

## Introduction

Aging leads to a decline in GFR, even in healthy adults and is an important risk factor for CKD.^1–3^ The decline in GFR is due to loss of glomeruli, predominately in the superficial cortex, that become ischemic appearing (global deflation and capsule thickening) before they undergo global sclerosis, atrophy, and are eventually reabsorbed.^4,5^ Global glomerulosclerosis occurs both with aging and with CKD. When global glomerulosclerosis exceeds age-specific normative thresholds, there is an increased risk of progressive CKD.^6^ Although disease-related glomerulosclerosis is diffused across cortical depths, age-related glomerulosclerosis occurs predominately in the superficial cortex. For example, among 85 year olds, about 33% of superficial and 13% of deep glomeruli are globally sclerosed, whereas among 30 year olds, about 2% of superficial and 3% of deep glomeruli are globally sclerosed.^7^ Thus, there is a need for more robust markers that can be used to characterize aging-related changes in the kidney at the molecular level to understand the processes that lead to glomerulosclerosis. Furthermore, biologic aging can occur at different rates and molecular markers may help identify patients with accelerated kidney aging at risk for adverse outcomes.^8^

Although senescence in glomeruli and in podocytes particularly has been studied in rodent models,^3^ molecular changes in the glomeruli with human aging remain less well understood. In particular, proteomic studies of kidney aging have focused on whole kidney tissue in rodent models,^9,10^ limiting insights into glomerulus-specific alterations. As glomeruli constitute only about 4% of the cortical parenchymal volume, analysis of whole kidney tissue would overlook key molecular changes specific to glomerular structures. Furthermore, analysis of whole kidney tissue comprises both sclerosed and nonsclerosed glomeruli. The proteome of sclerosed glomeruli represents the end result of a process readily detectable on standard histology. To gain insights into the early molecular processes of glomerular aging, the proteome of normal-appearing glomeruli on standard histology warrants study.

In this study, we used laser capture microdissection (LCM) to specifically sample nonischemic appearing, nonsclerosed glomeruli from the superficial cortex of both young and old adults who underwent a radical nephrectomy for a kidney tumor and in the absence of any CKD. We performed an unbiased and in-depth label-free quantitative proteomic analysis to characterize molecular differences in the glomeruli associated with aging. Our primary objective was to discover and then validate proteins that were differentially expressed in normal-appearing glomeruli of the superficial cortex in kidneys from old adults compared with young adults. Our secondary objective was to determine if these differentially expressed proteins were associated with kidney structural and functional pathology through age-independent pathways.

## Methods

### Study Design

The study participants were identified from a published cohort of Mayo Clinic Nephrectomy Registry patients in the Aging Kidney Anatomy study.^7^ The data were obtained with Mayo Clinic Institutional Review Board approval and waiver of consent as data were limited to medical records and tissue specimens obtained for clinical care. From 2000 to 2019, 1608 patients who had a radical nephrectomy for a kidney tumor with no metastatic lesions or positive lymph nodes at the time of surgery were identified. As previously described, a large wedge section of kidney parenchyma at least 2 cm away from the tumor-involved tissue was cut from the formalin-fixed whole-kidney specimens and embedded in paraffin.^11^ Formalin fixation and paraffin embedding (FFPE) was performed using standard operating procedures. The proteome profiles of the FFPE tissues have been reported to be relatively stable regardless of the preservation duration of FFPE blocks.^12^ Digital images of periodic acid-Schiff-stained 3-*µ*m thick histologic sections from the tissue block were reviewed and annotated, as described below. Those with histologic evidence of a diffuse specific kidney disease (other than mild-moderate diabetic nephropathy), >5% interstitial fibrosis and tubular atrophy (IFTA) by morphometry, and >5% inflammation by morphometry were excluded. The discovery dataset was initially defined by all 36 patients younger than 40 years and a random sample of 36 patients older than 70 years. After reviewing kidney wedge sections, three were excluded for inadequate number of glomeruli for proteomic analysis, leaving 34 old and 35 young adults (Supplemental Figure 1). Owing to limited availability of young individuals, the independent validation dataset included only six patients who were younger than 50 years and an additional eight patients older than 70 years (Supplemental Figure 1).

### Clinical, Histologic, and Imaging Measures

Baseline age, sex, body mass index, prenephrectomy serum creatinine (corrected to standardized values if assayed before standardization), 24-hour urine protein, hypertension, diabetes mellitus, and tobacco smoking were obtained from the medical records of patients with renal tumors. The eGFR was calculated using the serum creatinine–based 2021 CKD Epidemiology Collaboration equation.^13^ A spot urine protein-osmolality ratio was used to calculate 24-hour urine protein excretion.^14^

To reduce bias, investigators evaluated the images of the periodic acid-Schiff–stained sections morphometrically while blinded to clinical factors. The scanned digital images were magnified with Image Scope software (version 12.4.3.7009 Aperio) onto a large touch-screen tablet. All nonsclerosed glomeruli, ischemic glomeruli, globally sclerosed glomeruli, IFTA foci, intimal and lumen layers of three most orthogonal small-medium arteries, and the cortex were annotated. From these annotations, the (nonsclerosed) glomerular volume, %globally sclerosed glomeruli (%GSG), %ischemic (nonsclerosed) glomeruli, %artery luminal stenosis from intimal thickening, %IFTA (area density), and IFTA foci density (count density) were calculated, as previously described.^11^ The %GSG was calculated in the most superficial cortex (3–4 glomerular diameters), whereas other histologic morphometry was based on the entire cortex.

A semiautomated image processing tool (ITK-SNAP software, version 2.2; University of Pennsylvania, Philadelphia, PA) was used to obtain kidney cortex volume and medulla volume of the unaffected kidney from the prenephrectomy computed tomography or magnetic resonance images, as previously reported.^15^ Nephron number was calculated from the product of this cortical volume and the estimated three-dimensional nonsclerosed glomerular density based on histology, as previously described.^4^

### LCM and Proteomic Analysis

**Figure 1:**
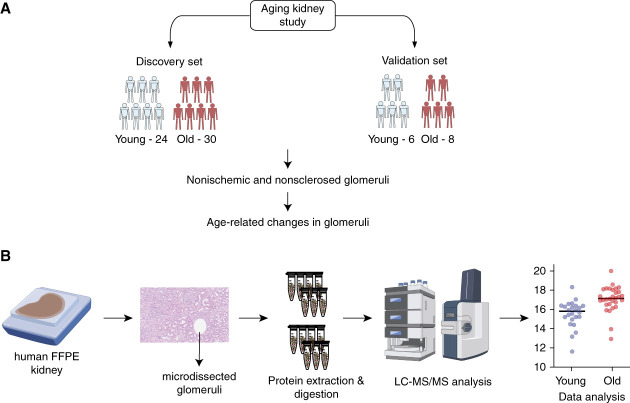
**Age-related changes in glomeruli isolated by LCM.** (A) Study design integrating discovery and validation datasets to investigate glomerular changes associated with aging. (B) Experimental workflow for label-free proteomic analysis of laser capture microdissected glomeruli. FFPE, formalin fixation and paraffin embedding; LCM, laser capture microdissection; LC-MS/MS, liquid chromatography–tandem mass spectrometry.

LCM was performed using the same FFPE kidney tissue blocks. Three consecutive sections were cut at a thickness of 10 *µ*m and mounted on polyethylene naphthalate membrane slides. In a random order, nonsclerosed, nonischemic healthy glomeruli from the superficial half of the cortex were collected targeting an area of at least 3 million *µ*m^2^ (Figure 1A) for subsequent proteomic analysis (Figure 1B). No significant difference in the number of glomeruli microdissected was observed between young and old adults (Supplemental Figure 2A). Mass spectrometry analysis was performed using timsTOF pro mass spectrometer (Bruker Daltonics, Bremen, Germany) connected to UltiMate 3000 RSLCnano in diaPASEF mode. Label-free quantitative analysis was performed using FragPipe software suite (version 21.1). Details regarding the proteomic analysis are described in the Supplemental Methods. Samples were excluded if the number of precursors identified was less than mean −1.5 times the SD of the number of precursors identified across the samples (<35,000). Overall, 30 old and 24 young adults were included for further analysis in the discovery dataset. The protein intensity data were normalized across the samples before performing the differential expression analysis (Supplemental Figure 2B). Eight old and six young adults were included for further analysis in the validation dataset.

### Statistical Analysis

Statistical analysis of mass spectrometry data was performed using Perseus computational platform.^16^ In brief, protein abundance values were log transformed, and proteins quantified in at least 70% within each age group (old or young) were included for further analysis. Missing values were imputed assuming a normal distribution with default width 0.3 and downshift 1.8 settings in Perseus software. Differential protein expression analysis between the young and old adults in the discovery set was performed using a two-sample two-sided *t* test with multiple hypothesis correction using permutation-based false discovery rate. Glomerular proteins that were significantly differentially expressed in the discovery dataset (q value <0.10) were assessed for differential expression between young and old adults in the validation set using a *t* test at a nominal *P* value < 0.05. Pathway enrichment analysis was performed using ShinyGO webserver v0.82,^17^ analyzing proteins with increased and decreased expression separately, with Reactome pathways as the reference database. Proteins that were detected and quantified in the discovery dataset were used as the background gene list. The glomerular proteins that were independently validated were assessed for their association with kidney pathology (eGFR, glomerular volume, %GSG, %ischemic glomeruli, %luminal stenosis, %IFTA, IFTA foci density, kidney cortex volume, kidney medulla volume, and nephron number) using Spearman rank correlation. Spearman partial correlations assessed correlations with kidney pathology after adjusting for age group (old versus young).

## Results

### Clinical and Histologic Characteristics

The mean age difference between the old and young adults was 40.7 years in the discovery dataset and 33.7 years in the validation dataset (Table 1). In the discovery dataset, old adults were more likely to have hypertension, lower eGFR, earlier tumor stage, smaller kidneys, more globally sclerosed glomeruli, more ischemic nonsclerotic glomeruli, more IFTA, more artery luminal stenosis from intimal thickening, and lower nephron number compared with young adults. Similar trends were observed in the validation dataset, although they were not statistically significant except eGFR and IFTA.

**Table 1:** Summary of nephrectomy patients selected for the discovery and validation sets

Characteristics	Discovery Set			Validation Set		
	Young (*N*=24)	Old (*N*=30)	*P* Value	Young (*N*=6)	Old (*N*=8)	*P* Value
**Demographics**						
Age, yr	36.4 (2.9)	77.1 (5)	—	46.3 (2.7)	80.0 (4.6)	—
Sex—male, *n* (%)	12 (50)	19 (63)	0.33	4 (67)	6 (75)	>0.99
**Clinical**						
Body mass index, kg/m^2^	32.3 (10.7)	28.5 (4.9)	0.42	29.1 (11.6)	28.7 (3.2)	0.41
Hypertension, *n* (%)	5 (21)	24 (80)	<0.001^a^	3 (50)	6 (75)	0.58
Diabetes mellitus, *n* (%)	1 (4)	1 (3)	0.87	1 (17)	0 (0)	0.43
Smoking history, *n* (%)	12 (50)	9 (30)	0.052	5 (83)	3 (38)	0.14
24-h urine protein, mg	299.2 (250.6)	258.2 (209.4)	0.71	82.0 (21.2)	224.2 (162)	0.43
Baseline eGFR, ml/min per 1.73 m^2^	71.4 (13)	45.5 (9)	<0.001^a^	56.7 (3.7)	45.3 (3.9)	0.004^a^
**Tumor stage**			0.020^a^			0.50
1	5 (20)	13 (44)		2 (33)	3 (37)	
2	8 (33)	1 (3)		3 (50)	1 (12)	
3	9 (37)	13 (43)		1 (17)	0	
Unknown	2 (8)	3 (10)		0	1 (12)	
**Kidney volumes, cm** ^**3**^						
Total volume	190.2 (54.9)	145.1 (33.6)	0.002^a^	186.8 (48.6)	157.2 (44.4)	0.54
Cortex volume	146.6 (42.3)	100.6 (25.5)	0.003^a^	135.0 (36.5)	106.6 (30.1)	0.66
Medulla volume	56.4 (16.4)	40.9 (10)	0.008^a^	51.8 (12.2)	41.4 (9.3)	0.18
**Microstructural**						
Glomerular volume, mm^3^	0.0029 (0.0010)	0.0028 (0.0008)	0.55	0.0024 (0.0005)	0.0024 (0.0009)	0.95
%GSG, %	1.4 (1.4)	10.4 (7.3)	<0.001^a^	7.8 (13.4)	13.4 (7.0)	0.059
%Ischemic glomeruli, %	0.5 (1.1)	2.3 (2.5)	<0.001^a^	1.5 (1.4)	2.5 (2.1)	0.44
%Artery luminal stenosis, %	30.4 (13.7)	57.0 (17.6)	<0.001^a^	43.9 (16.1)	56.3 (16.7)	0.23
%IFTA, %	0.6 (0.9)	1.5 (1.0)	<0.001^a^	0.7 (0.9)	3.4 (3.7)	0.14
IFTA foci density, per cm^2^	7.7 (8.7)	35.6 (18.4)	<0.001^a^	11.3 (10.1)	40.5 (28.4)	0.02^a^
Nephron number (in thousands)^b^	1143.3 (335.3) (*n*=21)	806.8 (304.8) (*n*=29)	<0.001^a^	1163.2 (311.1) (*n*=5)	977.3 (307.2) (*n*=6)	0.36

Data are presented as mean (SD) or %. GSG, globally sclerosed glomeruli; IFTA, interstitial fibrosis and tubular atrophy.

a Statistically significant correlations.

b Sample size smaller due to missing data.

### Quantitative Proteomic Analysis of Glomeruli in the Discovery Dataset

**Figure 2:**
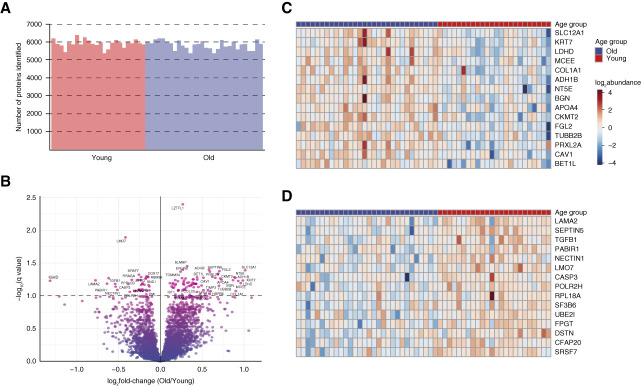
**Analysis of protein expression differences in glomeruli with age.** (A) Summary of the number of proteins identified in mass spectrometry analysis of glomeruli across young and old adults in discovery dataset. (B) Volcano plot indicating differential expression of proteins in old adults compared with young adults. Proteins with an adj. *P* value < 0.1 (indicated by dashed line) were considered significantly differentially expressed. A representative set of differentially expressed proteins were labeled. (C) Heatmap visualization of the top overexpressed proteins in old adults compared with young adults. (D) Heat map visualization of the top under expressed proteins in old adults compared with young adults. Each row corresponds to a protein represented by nonitalic gene symbol as indicated on the right side of the heat map. APOA4, apolipoprotein A-4; CASP3, caspase-3; COL1A1, collagen *α*-1(I) chain; KRT7, keratin type 2 cytoskeletal 7; LAMA2, laminin subunit *α*-2; LDHD, D-lactate dehydrogenase; LMO7, LIM domain only protein 7; MCEE, methylmalonyl-CoA epimerase; NT5E, Ecto-5′-nucleotidase; SEPTIN5, septin-5; SLC12A1, solute carrier family 12 member 1.

A mean of 144 nonischemic, nonsclerosed glomeruli were individually dissected for each patient. Overall, a median of 5877 proteins was identified across the 54 adults in the discovery dataset (Figure 2A). Differential expression analysis was performed on 5563 proteins that were quantified in at least 70% samples in each group (Supplemental Table 1). In total, 114 proteins were significantly differentially expressed (q value <0.1): 75 proteins with increased expression and 39 proteins with decreased expression in old adults compared with young adults (Figure 2B and Supplemental Table 2). Solute carrier family 12 member 1, keratin type 2 cytoskeletal 7, probable D-lactate dehydrogenase, methylmalonyl-CoA epimerase, and collagen *α*-1(I) chain were among the top proteins with increased expression in glomeruli from old adults (Figure 2C). Similarly, laminin subunit *α*-2, septin-5, TGF-*β*1 proprotein, LIM domain only protein 7, and caspase-3 were among the top proteins with decreased expression (Figure 2D).

### Characterization of the Differentially Expressed Proteins in Old Adults

**Figure 3:**
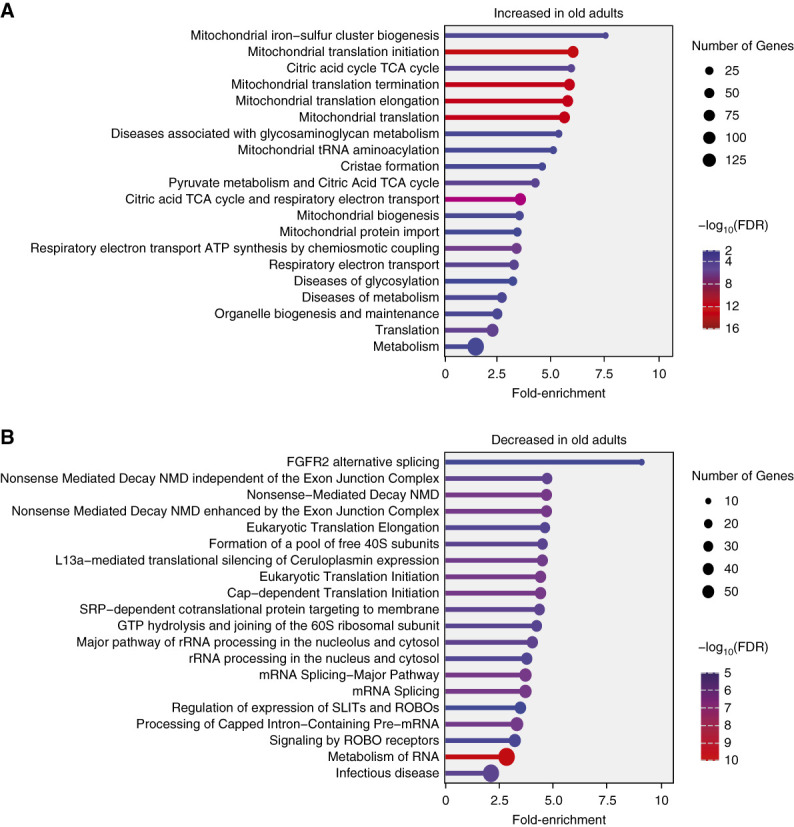
**Functional characterization of the differentially expressed proteins identified in the discovery dataset.** (A) Lollipop plot indicating the significant enrichment of Reactome pathway terms among the proteins with increased expression in old adults compared with young adults in discovery dataset. (B) Lollipop plot indicating the significant enrichment of Reactome pathway terms among the proteins with decreased expression in old adults. Proteins that were significantly differentially expressed with *P* value ≤ 0.05 and a fold-change of ±0.1-fold were considered for the analysis. Enrichment analysis was performed in ShinyGO v0.82 webserver using all the proteins identified in the discovery dataset as a background gene list. FDR, false discovery rate; NMD, nonsense-mediated decay.

Pathway enrichment analysis of the differentially expressed proteins identified in the discovery set was performed. There was significant enrichment of mitochondrial processes among the proteins with increased expression in old adults such as mitochondrial translational processes, aerobic respiration, and TCA cycle (Figure 3A). Proteins that are involved in these processes include ribosomal protein L11 (MRPL11), ribosomal protein L18 (MRPL18), ribosomal protein L15 (MRPL15), ribosomal protein L12 (MPRL12), and *α*-ketoglutarate dehydrogenase component 4 (MRPS36) that code for different mitochondrial ribosomal subunits, aconitate hydratase, succinate dehydrogenase complex flavoprotein subunit, succinate dehydrogenase cytochrome b560 subunit, isocitrate dehydrogenase (NADP) involved in TCA cycle and cytochrome c oxidase subunit 5B, NADH dehydrogenase (ubiquinone) 1 *α* subcomplex subunit 10, NADH dehydrogenase (ubiquinone) iron-sulfur protein 6, electron transfer flavoprotein subunit *α*, and electron transfer flavoprotein subunit *β* involved in electron transport chain (Supplemental Table 3). Protein with decreased expression in old adults was enriched in RNA metabolic processes such as mRNA splicing, mRNA processing, and non–sense-mediated decay (Figure 3B). The proteins involved in non–sense-mediated decay include nuclear cap-binding protein subunit 1, polyadenylate-binding protein 1, RNA-binding protein with serine-rich domain 1, and FAU ubiquitin-like and ribosomal protein S30 (Supplemental Table 4).

### Validation of the Candidate Proteins Identified in the Discovery Dataset

**Figure 4:**
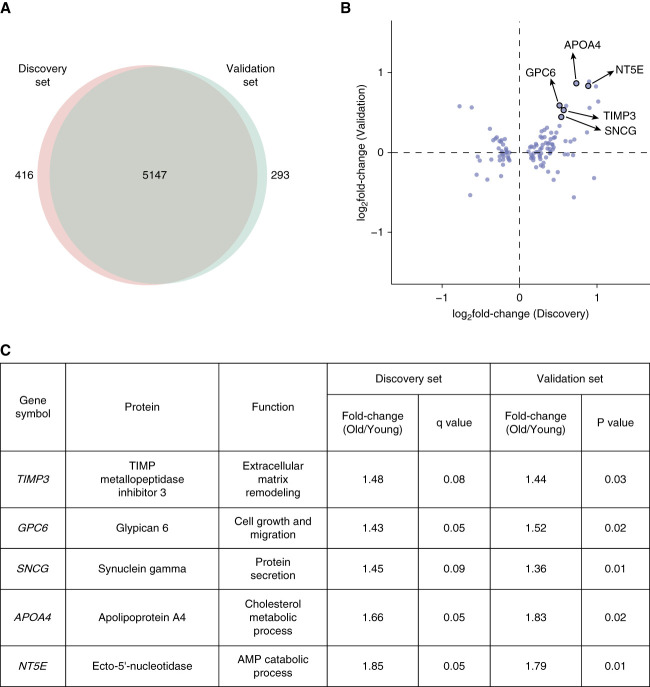
**Validation of the differentially expressed proteins identified from the discovery set.** (A) Overlap of the number of proteins identified in both discovery and validation datasets. (B) Scatter plot of fold-change values of proteins in the discovery and validation datasets. Only significantly differentially expressed proteins identified in the discovery dataset were represented in the scatter plot. Black border around the circle indicates statistically significant fold-change value in the validation dataset. (C) Table depicting the proteins that are significantly differentially expressed in both the discovery and validation datasets. ECM, extracellular matrix.

An independent dataset comprising six young and eight old adults was used to validate the differentially expressed proteins identified from the discovery analysis. Proteomic analysis was performed using the same workflow used for the discovery dataset. A total of 5147 proteins were consistently quantified across both the discovery and validation datasets, indicating robustness of the proteomic analysis (Figure 4A). We then assessed the expression of the differentially expressed proteins identified from the discovery dataset (Figure 4B). In common, five proteins, *i.e*., tissue inhibitor of metalloproteinase-3 (TIMP3), glypican 6 (GPC6), synuclein *γ* (SNCG), apolipoprotein A-4 (APOA4), and Ecto-5′-nucleotidase (NT5E) were identified with significantly increased expression in the old adults in the validation dataset at a *P* value < 0.05 (Figure 4C).

### Correlation of Validated Differentially Expressed Proteins in Glomeruli by Age with Kidney Pathology

We performed a Spearman correlation analysis for TIMP3, GPC6, SNCG, APOA4, and NT5E to assess their correlation with kidney pathology using the combined discovery and validation datasets (*n*=68). Increased expression of these proteins was significantly correlated with lower eGFR, higher %GSG, and higher IFTA foci density (Table 2). Some of these proteins also correlated with smaller cortical volume, smaller medulla volume, more ischemic glomeruli, more artery luminal stenosis, and higher %IFTA. None correlated with glomerular volume. We then performed the correlation analysis adjusting for old versus young age group (Table 2). None of the proteins showed significant correlation with kidney pathology independent of age group.

**Table 2:** Spearman correlations of kidney pathology with differentially expressed proteins

Kidney Pathology	Spearman Correlation (*P* Value)				
	TIMP3	APOA4	SNCG	NT5E	GPC6
Baseline eGFR	−0.37 (0.003)^a^	−0.33 (0.009)^a^	−0.43 (<0.001)^a^	−0.28 (0.02)^a^	−0.31 (0.01)^a^
Cortex volume	−0.18 (0.29)	−0.36 (0.031)^a^	−0.40 (0.02)^a^	−0.29 (0.09)	−0.11 (0.51)
Medulla volume	−0.19 (0.27)	−0.38 (0.02)^a^	−0.31 (0.07)	−0.24 (0.15)	−0.20 (0.23)
Glomerular volume	−0.17 (0.35)	−0.01 (0.96)	−0.02 (0.88)	0.07 (0.54)	0.07 (0.56)
%GSG	0.27 (0.02)^a^	0.37 (0.002)^a^	0.46 (<0.001)^a^	0.35 (0.003)^a^	0.37 (0.002)^a^
%Ischemic glomeruli	0.18 (0.13)	0.16 (0.18)	0.19 (0.12)	0.41 (0.001)^a^	0.33 (0.006)^a^
%Artery luminal stenosis	0.21 (0.09)	0.32 (0.007)^a^	0.34 (0.004)^a^	0.33 (0.006)^a^	0.19 (0.12)
%IFTA	0.19 (0.12)	0.13 (0.27)	0.24 (0.05)	0.40 (0.001)^a^	0.44 (<0.001)^a^
IFTA foci density	0.30 (0.01)^a^	0.27 (0.02)^a^	0.38 (0.001)^a^	0.44 (<0.001)^a^	0.48 (<0.001)^a^
Nephron number	−0.38 (0.002)^a^	−0.33 (0.01)^a^	−0.26 (0.04)^a^	−0.26 (0.05)^a^	−0.13 (0.33)

Kidney Pathology	Age Group-Adjusted Partial Spearman Correlation (*P* Value)				
	TIMP3	APOA4	SNCG	NT5E	GPC6
Baseline eGFR	−0.15 (0.24)	−0.06 (0.64)	−0.11 (0.40)	0.13 (0.32)	0.05 (0.70)
Cortex volume	0.02 (0.93)	−0.26 (0.13)	−0.18 (0.30)	−0.05 (0.76)	0.16 (0.37)
Medulla volume	−0.001 (0.99)	−0.29 (0.09)	−0.07 (0.69)	−0.01 (0.95)	0.03 (0.88)
Glomerular volume	−0.09 (0.45)	0.03 (0.80)	0.02 (0.84)	0.14 (0.27)	0.13 (0.30)
%GSG	−0.06 (0.64)	0.05 (0.67)	0.11 (0.36)	−0.08 (0.51)	−0.01 (0.92)
%Ischemic glomeruli	−0.02 (0.85)	−0.10 (0.41)	−0.12 (0.33)	0.19 (0.12)	0.1 (0.44)
%Artery luminal stenosis	−0.04 (0.75)	0.08 (0.52)	0.05 (0.66)	0.02 (0.85)	−0.15 (0.23)
%IFTA	−0.06 (0.62)	−0.18 (0.15)	−0.09 (0.45)	0.13 (0.28)	0.20 (0.1)
IFTA foci density	0.005 (0.97)	−0.10 (0.42)	0.003 (0.98)	0.09 (0.46)	0.19 (0.12)
Nephron number	−0.22 (0.09)	−0.13 (0.30)	0.02 (0.86)	0.02 (0.86)	0.22 (0.1)

All correlation values were round off to two decimals.

All *P* values were round off to two decimals if >0.01, or to three decimals if <0.01. APOA4, apolipoprotein A-4; GPC6, glypican 6; GSG, globally sclerosed glomeruli; IFTA, interstitial fibrosis and tubular atrophy; NT5E, Ecto-59-nucleotidase; SNCG, synuclein g; TIMP3, tissue inhibitor of metalloproteinase-3.

a Proteins with significant correlations.

## Discussion

Nephrosclerosis and nephron loss occurs with aging and is characterized by ischemic appearing glomeruli and glomerulosclerosis in the more superficial kidney cortex.^7^ We hypothesized that normal-appearing glomeruli might have molecular changes with age that are not appreciated on routine histology. Thus, in contrast to published studies, we investigated spatially localized normal-appearing glomeruli from the superficial cortex of young and old adults by proteomic analysis. This enabled us to uncover the protein alterations that were associated with aging in glomeruli before ischemic appearing changes or sclerosis were evident. In the initial discovery set, we identified increased expression of proteins that were previously reported to be associated with aging such as TIMP3,^18^ ADIRF,^19^ and CAV1.^20^ Notably, a recent study showed an increased expression of CAV1 in renal cortex of aging mouse model highlighting its role in promoting senescence by modulating AMPK-mTOR signaling pathway.^21^

Pathway enrichment analysis revealed increased expression of proteins involved in mitochondrial functions to be associated with aging. Mitochondrial dysfunction with aging has been attributed to increased oxidative stress, accumulation of mutations in mitochondrial DNA, and reduced clearance of damaged mitochondria due to impaired autophagy.^22,23^ An increase in mitochondrial size and mass has been observed as a common feature of aged senescent cells, suggesting a compensatory response to a decline in functional mitochondria.^22^ Since mitochondria are crucial to maintaining podocyte homeostasis and glomerular filtration,^24^ increased expression of mitochondrial ribosomal proteins and proteins involved in metabolic processes in normal-appearing nonsclerosed glomeruli of old adults potentially indicates an increased mitochondrial biogenesis as a compensatory mechanism to mitochondrial loss from oxidative stress. In addition, we also observed differences in RNA metabolic processes to be associated with aging. RNA binding proteins participate in regulating the post-transcriptional gene expression by modulating their stability, localization, and degradation. Dysregulation of these proteins has been associated with senescence and aging.^25^

We were able to validate the increased expression of five proteins in old adults using a validation set: TIMP3, GPC6, SNCG, APOA4, and NT5E. TIMP3 belongs to a family of tissue inhibitors of metalloproteases that plays an important role in extracellular matrix remodeling by regulating the activity of matrix metalloproteinases. Consistent with this study, expression of TIMP3 has been positively correlated with aging across different tissues including kidney.^26,27^ Loss of TIMP3 expression in a mouse model led to tubular and glomerular defects, followed by tubulointerstitial injury and renal fibrosis suggesting a protective role of TIMP3 in kidney.^28^ Expression of tissue inhibitors of metalloproteases family members including TIMP3 was also observed at advanced stages of replicative senescence in primary fibroblasts^29^; however, its exact role in senescence is not known. GPC6 is another extracellular matrix protein that was increased in old adults. It is a member of the glypican family, a group of cell surface proteoglycans that are attached to the plasma membrane through a glycosylphosphatidylinositol anchor and play a role in cell growth and differentiation.^30^ Similar to TIMP3, increased levels of GPC6 were also associated with senescence in human pulmonary fibroblasts treated with doxorubicin.^31^

SNCG, along with synuclein *α* (SNCA), belong to synuclein family of proteins. Although these proteins play a role in neuronal function,^32^ they are also implicated in non-neuronal disorders including cancer.^33–35^ Notably, SNCA deletion in renal proximal tubule epithelial cells led to increased profibrotic gene expression, interstitial matrix deposition, and myofibroblast activation.^36^ Furthermore, its levels were decreased in mouse model of renal fibrosis and in patients with CKD, indicating a protective role of SNCA against renal injury.^36^ Analogous to the increased levels of SNCG in glomeruli from old adults observed in this study, levels of SNCG, along with SNCA, were also increased with aging in astrocytes in mouse brain.^37^ APOA4 is a component of chylomicrons involved in lipid transport. Although the role of APOA4 in glomeruli is not well known, increased plasma levels of APOA4 levels were reported in renal disease patients, and it was identified to be significant predictor of progression to kidney failure.^38–40^

NT5E, an enzyme which catalyzes the hydrolysis of AMP to adenosine, was significantly increased in old adults. Elevated adenosine levels along with NT5E and adenosine receptor (ADORA2B) expression were reported in a diabetic rat model,^41^ a hypertensive CKD mouse model and human kidneys from patients with CKD with or without hypertension.^42^ In addition, adenosine levels regulate NT5E and ADORA2B expression through a positive feedback loop by inducing hypoxia-inducible factor-1*α*.^42^ As NT5E plays a critical role in promoting lung^43^ and hepatic fibrosis,^44^ its expression was positively regulated by stimulation with TGF-*β*, a key mediator of fibrosis, in renal proximal tubular epithelial cells.^45^

There are potential limitations to these findings. The datasets that were used were all limited to patients who underwent a radical nephrectomy for a tumor, typically renal cell cancer. Thus, it is possible that these findings may not be representative of average or typical aging. However, access to an adequate number of glomeruli for mass spectrometry analysis is not feasible from other living human patient populations where only needle biopsies are obtained. Analyses were limited to comparing old adults to young adults as categories rather than age continuous due to the limited sample size that was feasible.

These findings suggest that aging is associated with several protein and pathway alterations in glomeruli that have a normal appearance on standard histology. Using independent datasets, we identified significantly increased expression of TIMP3, GPC6, SNCG, APOA4, and NT5E associated with aging glomeruli. In addition, differentially expressed proteins involved in mitochondrial biogenesis and RNA metabolic processes were associated with aging glomeruli. Further studies are needed to understand the functional role of these proteins in aging and their potential role in CKD. Targeted interventions based on the pathophysiology of these glomerular proteins have the potential to treat the earliest pathways in the kidney aging process.

## Supplementary Material

SUPPLEMENTARY MATERIAL

## Data Availability

Original data generated for the study are or will be made available in a public access repository upon publication. Data Type: Raw Data/Source Data. Repository Name: PRoteomics IDEntifications database. Linkable Citation: https://www.ebi.ac.uk/pride/login. The mass spectrometry proteomics data have been deposited to the Proteome Xchange Consortium *via* the PRoteomics IDEntifications partner repository with the dataset identifier PXD061392.

## Supplemental Material

This article contains the following supplemental material online at http://links.lww.com/KN9/B307, http://links.lww.com/KN9/B308, http://links.lww.com/KN9/B309, http://links.lww.com/KN9/B310, http://links.lww.com/KN9/B311.

Supplemental Methods

Supplemental Figure 1. Flow chart outlining the patient selection criteria for proteomic analysis in the discovery set.

Supplemental Figure 2. (A) Number of glomeruli laser capture microdissected from the superficial cortex of kidney tissue per individual. (B) Box plots of the normalized protein intensities across all the samples analyzed by mass spectrometry in the discovery dataset.

Supplemental Table 1. List of proteins identified in young and old adults in the discovery dataset.

Supplemental Table 2. List of differentially expressed proteins along with the fold-change and q value identified in the discovery dataset.

Supplemental Table 3. List of Reactome pathways enriched among significantly overexpressed proteins in old adults compared with young adults in the discovery dataset.

Supplemental Table 4. List of Reactome pathways enriched among significantly under expressed proteins in old adults compared with young adults in the discovery dataset.
