# Supplementary material for: Spatial Mass Spectrometry-Based Proteomic Analysis of Normal-Appearing Glomeruli from Young and Old Adults
Source: Kidney360. 2025 Sep 30;6(12):2071–81. doi: 10.34067/KID.0000000986 (PMC12708404; doi:10.34067/KID.0000000986)

Supplemental Material

Table of contents:

Supplemental methods

Supplemental Figure 1: A flow chart illustrating the criteria used to select young and old adults for proteomic analysis in both the discovery and validation sets.

Supplemental Figure 2: A. Number of glomeruli laser capture microdissected from the superficial cortex of kidney tissue per individual. **B.** Box plots of the normalized protein intensities across all the samples analyzed by mass spectrometry in the discovery dataset.

Supplemental Table 1: List of proteins identified in young and old adults in the discovery dataset.

Supplementary Table 2: List of differentially expressed proteins along with the fold-change and q-value identified in the discovery dataset.

Supplemental Table 3: List of Reactome pathways enriched among significantly overexpressed proteins in old adults compared to young adults in the discovery dataset.

Supplemental Table 4: List of Reactome pathways enriched among significantly under expressed proteins in old adults compared to young adults in the discovery dataset.

Supplemental methods

Sample processing for discovery and validation datasets

Laser capture microdissected glomeruli were collected in 35 µL of MS-grade water. To these samples, 65 µL of lysis buffer (300 mM Tris pH 8.2/0.005% Zwittergent 3-16) was added. Protein extraction and reduction with 5 mM TCEP was performed by heating samples at 98°C for 90 minutes and the extracted protein was subjected to sonication (BioRuptor Pico, Diagenode) for 20 minutes. Thereafter the sample tubes were transferred to the Hamilton liquid handling platform for alkylation (20 mM IAA for 20 minutes in dark) and overnight trypsin digestion at 37°C. Samples were acidified the next day and transferred from tubes to plate for mass spectrometry analysis.

For the validation dataset, laser capture microdissected glomeruli were sampled in MS-grade water and adjusted to a final concentration of 100 mM Tris pH 8.2/2% SDS/0.005% Zwittergent 3-16. Protein extraction and reduction with 5 mM TCEP was performed by heating samples at 98°C for 90 minutes and the extracted protein was subjected to sonication (BioRuptor Pico, Diagenode) for 20 minutes. After alkylation with 20 mM IAA for 20 minutes in dark, Sera-mag SpeedBeads were added in a 1:50 ratio (protein:beads) along with acetonitrile. These were then incubated for 10 minutes followed by two washes with 80% ethanol and one wash with 100% acetonitrile. These were then left for overnight trypsin digestion at 37°C. Digested peptides were collected, acidified and stored at -80°C for mass spectrometry analysis.

Spectral library construction

A pool of peptides from patients in the discovery dataset was used for constructing a spectral library. Peptide pool was fractionated using basic pH reversed-phase HPLC system on an UltiMate 3000 HPLC system (Thermo Fisher Scientific, Waltham, MA). Briefly, peptides were dissolved in

solvent A (5 mM ammonium formate, pH 9.0) and fractionated on a C_{18} XBridge column (1.7 µm, 150 x 1 mm, Waters) using increasing gradient of solvent B (5 mM ammonium formate, pH 9.0, 90% acetonitrile). The absorbance of eluted peptides was measured at 280 nm. The total run time was 90 min, 72 fractions were collected and pooled into 12 fractions for mass spectrometry analysis.

Mass spectrometry data acquisition and analysis

Mass spectrometry analysis was performed using timsTOF pro mass spectrometer (Bruker Daltonics, Bremen, Germany) connected to UltiMate 3000 RSLCnano system either in DDA-PASEF mode for spectral library generation or diaPASEF mode for quantitative analysis. Indexed retention time (iRT) synthetic standard peptides (Biognosys) were spiked into each sample before subjecting it to mass spectrometry analysis. Peptides were initially loaded on a trap column (Halo C_{18} 2.7 µm EXP2 stem trap, Optimize Technologies) using solvent A (0.1% formic acid). Gradient separation was performed using 3 to 28% solvent B (80% acetonitrile, 0.1% formic acid) on an analytical column (25 cm x 75 µm, 1.7 µm C_{18}, IonOpticks) maintained at 50°C for a total run time of 90 min. In DDA-PASEF mode, precursor ions were analyzed in PASEF mode with an ion mobility range of 0.6 to 1.6 Vs cm^{-2}, TIMS accumulation time and ramp time of 100 ms each leading to 100% duty cycle. MS/MS data were acquired with 10 PASEF MS/MS scans per cycle. Isolation width was set to 2 m/z for m/z < 700 and 3 m/z for m/z > 800. Collision energy was increased stepwise from 20 to 59 eV as a function of increasing ion mobility from 0.6 to 1.6 Vs cm^{-2}. MS and MS/MS spectra were acquired from m/z 100 to 1700.

In diaPASEF mode, variable isolation widths were generated based on the precursor densities in the spectral library using py_diAID algorithm. diaPASEF scans was carried out with two ion

mobility windows ranging 0.7-1.43 Vs cm^{-2} and mass range of 400−1,200 m/z. A total of 16 windows were analyzed with a total cycle of 1.8 seconds. The smallest DIA window size is 15 m/z and the largest DIA window is 100 m/z. Accumulation and ramp times were specified to 100 ms per scan. MS/MS fragmentation was performed using a linear ramp of collision energy from 20 to 59 eV for 1/K0 = 0.6 Vs cm^{-2} to 20 eV at 1/K0 = 1.6 Vs cm^{-2}. Ion mobility and mass calibration was performed using a filter spiked in with three ESI Tuning Mix ions (m/z, 1/K0: 622.02, 0.98 Vs cm^{-2}, 922.01, 1.19 Vs cm^{-2}, 1221.99, and 1.38 Vs cm^{-2}).

diaPASEF raw data was analyzed in FragPipe computation platform (version 21.1) using default settings of DIA_SpecLib_Quant workflow. Database searching was performed against Human UniProt protein database by MSFragger (version 4.0) using the settings: precursor and fragment ion tolerance of 20 ppm, strict trypsin cleavage with one missed cleavage, oxidation (methionine) and acetylation (protein N-terminus) as variable modifications and carbamidomethylation (cysteine) as static modifications. PSM validation was performed using MSBooster-based deep learning rescoring followed by Percolator algorithm. Philosopher (version 5.1) node was used to filter for protein and peptide identifications at 1% false discovery rate. EasyPQP was used to generate the spectral library based on the DDA-PASEF files. Finally, DIANN module (version 1.8.2) was used for protein quantitation from the diaPASEF raw files based on the spectral library.

Supplemental Figure 1

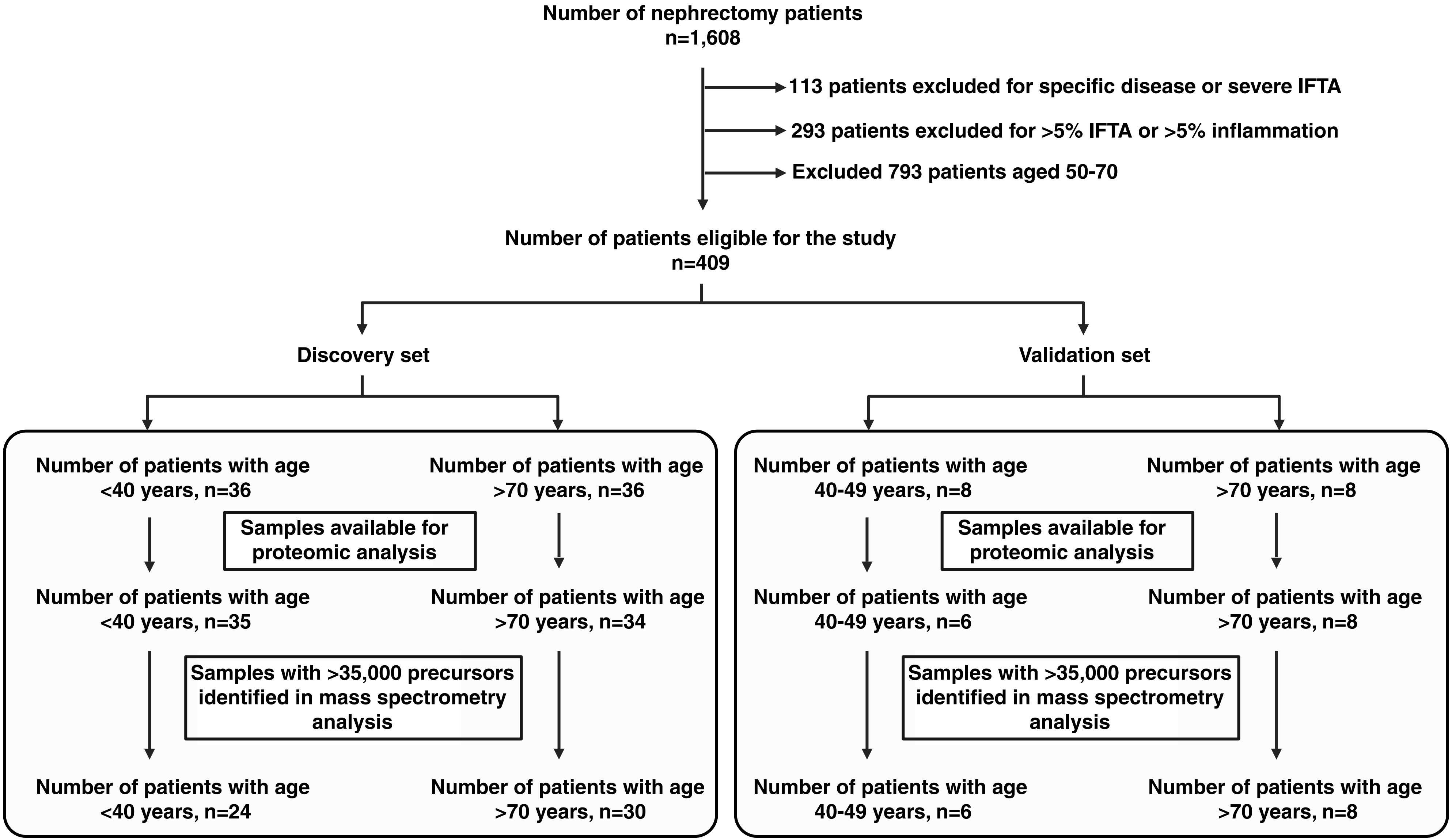

Supplemental Figure 2

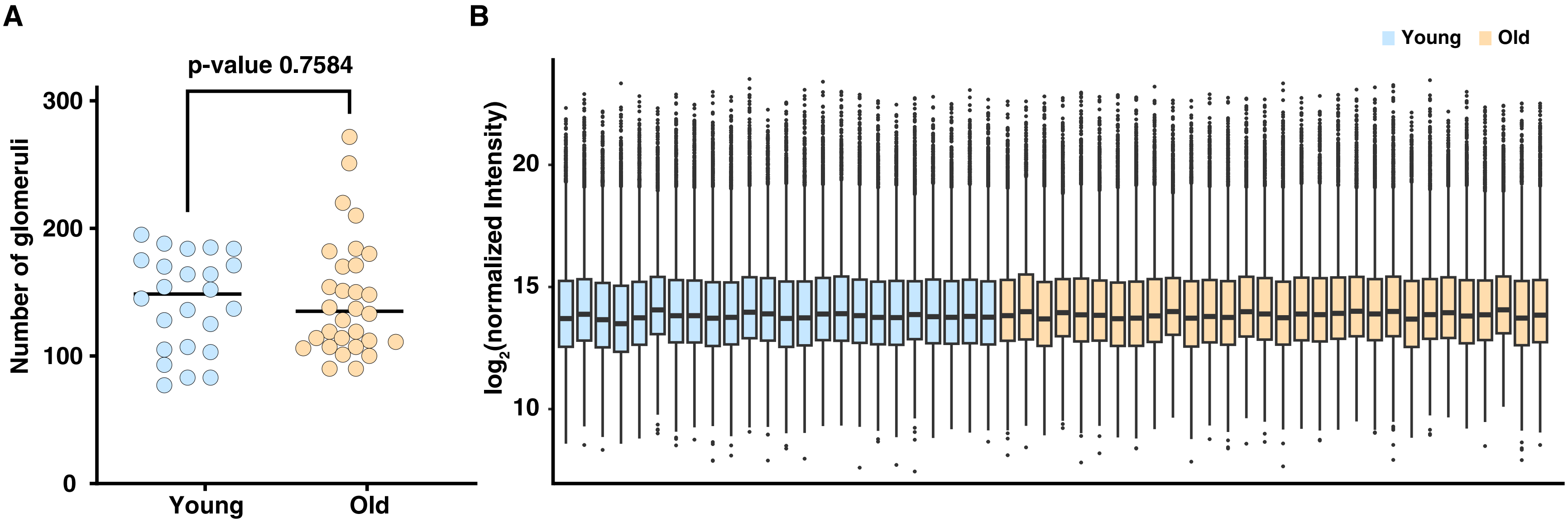

Supplement: SUPPLEMENTARY MATERIAL [file kidney360-6-2071-s002.pdf]
